# Catch Crop Residues Stimulate N_2_O Emissions During Spring, Without Affecting the Genetic Potential for Nitrite and N_2_O Reduction

**DOI:** 10.3389/fmicb.2018.02629

**Published:** 2018-11-02

**Authors:** Yun-Feng Duan, Sara Hallin, Christopher M. Jones, Anders Priemé, Rodrigo Labouriau, Søren O. Petersen

**Affiliations:** ^1^Department of Agroecology, Aarhus University, Tjele, Denmark; ^2^Department of Forest Mycology and Plant Pathology, Swedish University of Agricultural Sciences, Uppsala, Sweden; ^3^Section of Microbiology, Department of Biology, University of Copenhagen, Copenhagen, Denmark; ^4^Department of Mathematics, Aarhus University, Aarhus, Denmark

**Keywords:** catch crop, fertilization, nitrous oxide emissions, denitrifier genes, N_2_O-reduction genes

## Abstract

Agricultural soils are a significant source of anthropogenic nitrous oxide (N_2_O) emissions, because of fertilizer application and decomposition of crop residues. We studied interactions between nitrogen (N) amendments and soil conditions in a 2-year field experiment with or without catch crop incorporation before seeding of spring barley, and with or without application of N in the form of digested liquid manure or mineral N fertilizer. Weather conditions, soil inorganic N dynamics, and N_2_O emissions were monitored during spring, and soil samples were analyzed for abundances of nitrite reduction (*nirK* and *nirS*) and N_2_O reduction genes (*nosZ* clade I and II), and structure of nitrite- and N_2_O-reducing communities. Fertilization significantly enhanced soil mineral N accumulation compared to treatments with catch crop residues as the only N source. Nitrous oxide emissions, in contrast, were stimulated in rotations with catch crop residue incorporation, probably as a result of concurrent net N mineralization, and O_2_ depletion associated with residue degradation in organic hotspots. Emissions of N_2_O from digested manure were low in both years, while emissions from mineral N fertilizer were nearly absent in the first year, but comparable to emissions from catch crop residues in the second year with higher precipitation and delayed plant N uptake. Higher gene abundances, as well as shifts in community structure, were also observed in the second year, which were significantly correlated to ${\text{NO}}_{3}^{-}$ availability. Both the size and structure of the nitrite- and N_2_O-reducing communities correlated to the difference in N_2_O emissions between years, while there were no consistent effects of management as represented by catch crops or fertilization. It is concluded that N_2_O emissions were constrained by environmental, rather than the genetic potential for nitrite and N_2_O reduction.

## Introduction

Global anthropogenic emissions of nitrous oxide (N_2_O), a potent greenhouse gas and ozone-depleting substance, constitute 6.7 Tg nitrogen (N) annually according to Ravishankara et al. (2009). Agriculture is the single largest source of these emissions, contributing an estimated 5.8 Tg N and 4.2–7.0 Tg N using bottom-up and top-down approaches, respectively (Del Grosso et al., 2008). These N_2_O emissions are, directly or indirectly, related to the application of mineral fertilizers and manure for crop production, and decomposition of crop residues (Davidson, 2009). Soil N losses outside the main growing season reduce the overall N use efficiency of a cropping system, and winter cover crops (catch crops) are increasingly used as a measure against N leaching (Aronsson et al., 2016). When catch crop residues decompose following spring incorporation, N_2_O emissions can be triggered (Flessa et al., 2002). Some studies indicate that the main source of N_2_O is heterotrophic denitrification (Chen et al., 2013; Li et al., 2016; Parkin et al., 2016), although at low oxygen tensions the contribution from nitrifier-denitrification may also be significant (Poth and Focht, 1985; Zhu et al., 2013). Understanding the mechanisms, by which catch crop residues enhance N_2_O emissions, may help to develop new management practices in order to prevent indirect N_2_O emissions from N leaching during winter from being replaced by direct emissions during spring.

Denitrification is mediated through a sequence of enzyme-catalyzed reactions, in which nitrate (${\text{NO}}_{3}^{-}$) is reduced via nitrite (${\text{NO}}_{2}^{-}$) and nitric oxide (NO) to N_2_O or N_2_ under anoxic conditions by a diverse group of microorganisms. The denitrification pathway is modular, meaning that all steps in the pathway are not necessarily performed by the same organisms (Graf et al., 2014). Therefore, the abundances of *nir* genes, encoding enzymes that convert nitrite (${\text{NO}}_{2}^{-}$) to nitric oxide (NO), and *nos* genes, encoding enzymes responsible for N_2_O reduction to N_2_, inform about the balance between genetic potential for N_2_O production and consumption within a denitrifying community. A causal relationship between denitrification gene abundance and N_2_O emissions has been shown in experiments, where the relative abundance of organisms with or without *nosZ* genes was manipulated (Philippot et al., 2011; Domeignoz-Horta et al., 2016). Under field conditions, denitrification gene abundances and N_2_O emissions are sometimes, but not always, correlated (Hallin et al., 2009; Morales et al., 2010; Wang et al., 2017). Gene pools may not always reflect rates of N_2_O emissions due to subsequent controls over gene transcription and enzyme activities (Philippot and Hallin, 2005; Wallenstein et al., 2006; Röling, 2007). Thus, gene abundances may reflect the genetic potential within the cropping system, rather than short-term response to dynamic environmental conditions.

Emissions of N_2_O associated with incorporation of (catch) crop residues can vary due to differences in plant C:N ratio or decomposability. Li et al. (2016) reported that leguminous catch crop residues resulted in net N mineralization and significant N_2_O emissions even at 40% water-filled pore space (WFPS), while ryegrass caused net N immobilization and much lower N_2_O emissions. While residue N availability is important for denitrifier activity and N_2_O emissions, especially if soil ${\text{NO}}_{3}^{-}$ availability is low, residue C decomposability is also critical by constituting a sink for oxygen (O_2_). Thus, residue decomposition may interact with soil water content in determining soil O_2_ status around organic hotspots. For example, Li et al. (2013) found that crop residues consistently increased N_2_O emissions at 30 and 60% WFPS, while at 90% WFPS the emissions were reduced by residue amendment, presumably because there was a shift in the N_2_O:N_2_ product ratio of denitrification due to more reducing conditions. Finally, when catch crop residue incorporation in spring is followed by N fertilization, there is a potential for interactions between the external N source and the decomposing residues, which may enhance denitrification (Frimpong and Baggs, 2010) and N_2_O emissions (Duan et al., 2017).

Our aim was to better understand the complex interactions between soil conditions, crop residues and N amendments during spring, and the response of nitrite- and N_2_O-reducing communities, since this understanding is a precondition for effective strategies to mitigate N_2_O emissions. For this purpose, we performed a 2-year field study in which N_2_O emissions were monitored during spring in a factorial experiment that involved rotations with or without catch crops, and with or without application of N as digested liquid manure or mineral fertilizer. By the end of each monitoring period in June, the soil was sampled to analyze the abundance of nitrite and N_2_O reduction genes, and the structure of the communities harboring these genes. We hypothesized (1) that N-rich fertilizer and catch crop residues would interact positively on N_2_O emissions; (2) that N_2_O emissions derived from mineral N would depend more on soil O_2_ status, and hence rainfall, than emissions derived from catch crop residues; and (3) that the abundance and composition of denitrifying communities would reflect the long-term effects of cropping system on metabolizable C and N availability.

## Materials and methods

### Long-term crop rotation field experiment

The study made use of a long-term crop rotation experiment, established in 1996, that is located at 56°30′N, 9°34′E in Western Denmark (Olesen et al., 2000). The sandy loam is classified as a Typic Hapludult and has 77.9% sand, 13.3% silt and 8.8% clay in the plow layer (0–25 cm soil depth). This depth interval further contains 23 g kg^−1^ soil organic carbon (SOC) and 1.8 g kg^−1^ total N, and it has a pH_CaCl2_ of 6.5, a cation exchange capacity of 12.3 meq 100 g^−1^, and an average bulk density of 1.35 g cm^−3^. Mean annual rainfall is 704 mm and mean annual air temperature 7.3°C.

Five different cropping systems were compared, representing systems with or without catch crops, and with or without N fertilization (Table 1). All systems had rotations with spring barley (*Hordeum vulgare)*, hemp (*Cannabis sativa*), pea (*Pisum sativum*)/barley, spring wheat (*Triticum aestivum)* and potato (*Solanum tuberosum*). All crops were represented each year in two fully randomized blocks. Where a catch crop was present before spring barley (+*CC*), this was a mixture of rye (*Secale cereale*), hairy vetch (*Vicia villosa*) and rapeseed (*Brassica napus*). Four of the five rotations were under organic management (*O4*), and the last rotation under conventional management (*C4*), where the identifiers *O4* and *C4* are used in accordance with previous studies from this long-term crop ration experiment (e.g., Chirinda et al., 2010; Brozyna et al., 2013).

**Table 1:** Cumulative N_2_O emissions during spring, N_2_O emission factors (EFs), and yield-scaled EFs of spring barley in five crop rotations. For calculation of EFs, the N_2_O emissions were corrected for background emissions in treatment *O4-CC-N* with no external N input. Significant differences between rotations within a year are indicated by lower-case letters, and differences between years within a rotation by capital letters.

**System^#^**	**Catch crop**	**N fertilizer^§^**	**Nitrous oxide flux, kg N ha**^**−1**^				**Emission factor**		**Yield-scaled EF, kg N**_**2**_**O-N kg**^**−1**^ **N in plant uptake**	
			**2011**		**2012**		**2011**	**2012**	**2011**	**2012**
Conventional	*−CC*	*+N*	0.27	bA	0.96	cB	0.000	0.007	0.000	0.005
Organic	*+CC*	*+N*	0.79	cA	1.39	dB	0.004	0.007	0.006	0.015
Organic	*+CC*	*−N*	0.8	cA	0.91	cA	0.017	0.023	0.006	0.011
Organic	*−CC*	*+N*	0.2	aA	0.28	bB	−0.001	0.001	−0.001	0.004
Organic	–*CC*	–*N*	0.25	abA	0.18	aA	NA	NA	NA	NA

*NA, Not applicable*.

# *Crop sequence (all rotations): spring barley (Hordeum vulgare); hemp (Cannabis sativa); pea (Pisum sativum)/barley; spring wheat (Triticum aestivum); potato (Solanum tuberosum). In +CC rotations, a catch crop consisting of a rye (Secale cereale), hairy vetch (Vicia villosa) and rapeseed (Brassica napus) mixture was established after all crops in the rotation except hemp*.

§ *In the conventional system, the N source was NPK fertilizer, while in the organic rotations the N source was anaerobically digested liquid manure (see text for details)*.

Field plots within each rotation were selected in which the main crop in the previous year was potato, and the main crop in the experimental years (2011 and 2012) was spring barley. A rotation with neither catch crop nor N fertilization was not represented in the basic design, and instead manure application was excluded from a 1.5 m strip of *O4-CC*+*N* plots, which represented the treatment *O4-CC-N*. In *O4* rotations, the N fertilizer was anaerobically digested liquid manure, which contained 3.6% dry matter (DM), 6.5 kg Mg^−1^ total N and 3.9 kg Mg^−1^ total ammonia-N (TAN) in 2011, and 2.6% DM, 8.2 kg Mg^−1^ total N and 5.4 kg Mg^−1^ TAN in 2012. The two organic rotations with manure application received 99.4 kg ha^−1^ TAN in 2011, and 132 kg ha^−1^ TAN in 2012. The conventional rotation received 120 kg ha^−1^ N in NPK 23-3-6 (%, by weight) fertilizer, with similar amounts of ammonium (${\text{NH}}_{4}^{+}$) and ${\text{NO}}_{3}^{-}$ in both years.

### Field operations

The amount of N returned to the soil through incorporation of above-ground catch crop biomass was estimated by cuts to 1 cm height in mid-November of 2010 and 2011, respectively. Total DM and N percentage of cuts were determined. In 2011, rotovation and plowing (with incorporation of catch crops where present) took place on 6 April, N fertilization on 12 April, and seeding on 19 April. In 2012, the rotovation and plowing took place on 4 April, N fertilization on 10 April, and seeding on 11 April. There were no further field operations during the N_2_O monitoring period. In early August of both years, the above-ground biomass (including spring barley and weeds) was cut to determine DM production and N uptake in harvested biomass.

### Nitrous oxide measurements

The dimensions of field plots were 12 × 15 m, with a 6 × 15 m harvest plot in the middle, and to each side sampling plots with dedicated 1 × 1 m microplots for experimental purposes. For the present study, two available microplots per field plot were randomly selected for monitoring of N_2_O emissions. Two-part static chambers were used with permanently installed stainless steel collars covering a 0.75 × 0.75 m area. The chambers (height 20 cm) of 4 mm white expanded PVC were vented and further equipped with a battery-powered fan for mixing of the chamber headspace during deployment. When chambers were deployed for flux measurements, gas samples (10 mL) were collected through a septum using a polypropylene syringe and hypodermic needle, and stored in evacuated 6 mL exetainer vials (Labco, Ceredigion, UK) for later analysis. Five gas samples were taken over the course of *c*. 2 h starting around 9:30, the first sample at the time of deployment.

In 2011, the N_2_O monitoring started immediately after tillage, and two N_2_O-flux measurement campaigns were conducted in the week between tillage and fertilization; then collars were temporarily removed for manure application and incorporation, and seeding. Since 2011 showed no significant N_2_O emissions prior to fertilization, the first N_2_O flux measurement campaign in 2012 took place on the day of seeding. Three N_2_O flux measurement campaigns were then carried out during the first week, followed by weekly campaigns until mid-June.

Nitrous oxide concentrations in the gas samples were determined using an Agilent 7890 GC system with a CTC CombiPal autosampler (Agilent, Nærum, Denmark). The gas chromatograph had a 2-m back-flushed pre-column with Hayesep P, and a 2-m main column with Porapak Q connected to an electron capture detector. The carrier gas was N_2_ at a flow rate of 45 mL min^−1^, and Ar-CH_4_ (95/5%) at a flow rate of 40 mL min^−1^ was used as make-up gas. Temperatures of injection port, column and detector were 80, 80, and 325°C, respectively.

### Soil sampling

From the time of N fertilization, and then weekly until the end of N_2_O monitoring, soil samples were collected adjacent to micro-plots used for N_2_O flux measurements. Ten subsamples (20 mm diameter, 0–20 cm depth) were taken from each field plot and pooled. Subsamples (10 g) were extracted in 1 M KCl and filtered extracts frozen at −20°C until analyzed for ${\text{NH}}_{4}^{+}$ and ${\text{NO}}_{3}^{-}$ concentrations by standard colorimetric methods (Keeney and Nelson, 1982). Gravimetric soil water content was determined by drying 10 g of soil for 24 h at 105°C. For each sampling day, soil WFPS and relative gas diffusivity were calculated using treatment specific measurements of dry bulk density (Chirinda et al., 2010). Relative gas diffusivity was calculated as (Moldrup et al., 2005):

$$\frac{D_{p}}{D_{0}}=\Phi^{2}{\left(\frac{\varepsilon }{\Phi }\right)}^{2+[\log(\varepsilon_{100}{}^{0.25})/\log(\varepsilon_{100}/\Phi )]},$$

where *D*_*p*_ and *D*_0_ are gas diffusivity in soil and air, respectively (m^2^ s^−1^), Φ is total porosity (m^3^ m^−3^ soil), ε is volumetric air content (m^3^ m^−3^ soil), and ε_100_ is volumetric air content at −100 cm H_2_O.

After the final N_2_O emission measurement campaign in June of each year, two 250 cm^3^ soil samples were collected from 0 to 10 cm depth for molecular analyses within each of the permanently installed collars used for N_2_O monitoring. These samples were sieved and mixed separately, and subsamples frozen at −20°C until DNA isolation.

### DNA isolation

Microbial genomic DNA was isolated from soil samples using Genomic Spin Kit (A&A Biotechnology, Gdynia, Poland) following a modified protocol. A 500-mg soil sample was added to a tube containing small glass beads, followed by 1 mL extraction buffer (A&A Biotechnology). Cells in the soil were lysed using a FastPrep instrument (MP Biomedicals, Solon, OH, USA) for 30 s at a speed of 5.5, followed by centrifugation at 14,000 × *g* for 1 min, and then the supernatant was transferred to a sterile 1.5-mL Eppendorf tube. Ammonium acetate (5 M) was added to the tube to a final concentration of 2 M, and the tube was incubated on ice for 5 min after vortexing. Then, the tube was centrifuged at 16,000 × *g* for 10 min at 4°C, and the supernatant was transferred to a 9-mL plastic tube. Two mL guanidine HCl (7 M) was added to the tube and mixed by vortexing, and then 900 μL of the mixture was transferred to a spin column and centrifuged at 14,000 × *g* for 15 s. After centrifugation, the catch tube was emptied, and the process was repeated with another 900 μL liquid until the entire sample had run through the spin column. Finally, the spin column was washed, and the DNA was eluted according to the manufacturer's instructions.

The extracts were analyzed by 1% (w/v) agarose gel electrophoresis, and the bands containing genomic DNA were cut out for DNA recovery using SpinPrep Gel DNA Kit (Millipore, Hellerup, Denmark). The quantities of extracted DNA were determined using Qubit dsDNA BR assays (Invitrogen, Carlsbad, CA, USA). After quantification, the DNA were diluted to 10 ng μL^−1^ and kept at −20°C until used for downstream analysis.

### Quantification of *nirK, nirS*, and *nosZ* genes

Quantitative real-time PCR (qPCR) was performed using a Bio-Rad CFX96 Real-Time System (Bio-Rad, Hercules, CA, USA). Prior to gene quantification, the presence of potential PCR inhibitors in each soil DNA extract was tested by quantifying a known amount of the pGEM-T plasmid (Promega, USA) using plasmid specific T7 and SP6 primers in the presence of extracted DNA or water. The 15 μL mixture for inhibition test contained 1 × DyNAmo Flash SYBR Green qPCR Master Mix (Thermo Scientific, Waltham, MA, USA), 1 μg bovine serum albumin (BSA; New England BioLabs, MA, USA), 0.25 μM of each primer, 1 × 10^5^ copies of the plasmid, and 2 μL of either soil DNA (20 ng) or water. No inhibition was observed with the amount of DNA used.

Standards ranging from 1 × 10^2^ to 10^8^ gene copies μL^−1^ were prepared from linearized pGEM plasmids with insertions of fragments of the target genes (*nirK, nirS, nosZ*-I, or *nosZ*-II). The genes *nirK* and *nirS* were amplified with primers F1aCu/R3Cu (Hallin and Lindgren, 1999) and Cd3aF/R3cd (Throbäck et al., 2004), respectively; and *nosZ* clades I and II were amplified using primers 1840F/2090R (Henry et al., 2006) and *nosZ*-II-F/*nosZ*-II-R, respectively (Jones et al., 2013). The 15 μL qPCR mixture consisted of 1 × DyNAmo Flash SYBR Green qPCR Master Mix, 1 μg bovine serum albumin, 0.25 μM (for *nirK*) or 0.8 μM (for *nirS* and *nosZ*) of each primer, and 2 μL (20 ng) of template. Primers and thermal cycling conditions are detailed in Table S1 in Supplementary Material. Each gene was amplified twice on two separate plates. Dissociation curve analysis and agarose gel electrophoresis of amplicons were performed at the end of each run to confirm the specificity of amplification. Amplification efficiencies were 90, 94, 98, and 85% for *nirK, nirS, nosZ*-I, and *nosZ*-II, respectively. Results were processed using Bio-Rad CFX Manager software version 3.1 with default settings.

### Terminal restriction fragment length (T-RFLP) analysis

PCR for T-RFLP analysis was performed on a Bio-Rad C1000 Thermal Cycler (Bio-Rad, Hercules, CA, USA). The same primers as those for qPCR were used for amplification, with the modification that the 5′ ends of the forward primers were labeled with the fluorescent dye hexachlorofluorescein (HEX). The 40-μL PCR mixture contained 20 μL DreamTaq Green PCR Master Mix (Thermo Scientific), 4 μg bovine serum albumin, 0.25 μM (for *nirK*) or 0.8 μM (for *nirS* and *nosZ*) of each primer, and 20 ng of soil DNA. The thermal cycling conditions were identical to those used for qPCR, with the modification of exclusion of the data acquisition step and the melting curve analyses. Amplicons were analyzed by agarose gel electrophoresis to confirm successful amplification and correctness of fragment sizes. Amplicons of each gene were digested by two different restriction endonucleases separately to produce terminal restriction fragments (T-RFs): *nirK* amplicons were treated by HaeIII and HpyCH4IV, *nirS* by HaeIII and HhaI, *nosZ*-I by BstUI and Sau96I, and *nosZ*-II by HpyCH4IV and NlaIII (all restriction enzymes were from New England BioLabs, Ipswich, MA, USA). Enzyme digestions were performed according to manufacturer's instructions. T-RFLP profiling was performed using a 3,730xl DNA Analyzer (Applied Biosystems, Waltham, MA, USA) at Uppsala Genome Center, Uppsala University, Sweden, and data on peak positions and sizes were extracted using the Peak Scanner software (Applied Biosystems).

T-RFs from different soil samples were aligned using an in-house R package (see Supplementary Material for R source code and analysis parameters), which uses tables of peak size, area, and height exported from Peak Scanner as input and aligns profiles in a series of steps. First, each profile was processed using a Gaussian smoothing function, which eliminated double or shoulder peaks by a peak merging algorithm. The peaks were then relativized by dividing peak areas and heights by the sum of each within the same profile. Next, peaks across all samples were differentiated into “noise” and “signal” peaks using the iterative approach described by Abdo et al. (2006), where noise peaks were defined as having relative areas or heights < 3 standard deviations from a theoretical baseline of 0 relative fluorescence units (RFUs) across all samples. After removal of noise peaks, signal peaks were aligned across all profiles using the iterative dynamic programming algorithm described by Vähämaa et al. (2007). Briefly, two de-noised T-RFLP profiles were selected at random and aligned in a pair-wise manner using dynamic programming, where dissimilarities between peaks in each profile account for differences in peak size as well as area and height. New profiles were then added to the alignment in random order using a modified version of the dynamic programming algorithm, where the set of aligned T-RFLP profiles were converted into single profiles of average peak size, area, and heights. Once all samples were aligned, an overall alignment score was calculated based on the sum of peak dissimilarities. Then followed an iterative process, where a sample is chosen at random and removed from the alignment, then realigned to the remaining samples. Once this was done for all samples, the overall alignment score was recalculated and, if the score was improved from the previous alignment, another iteration was carried out using the improved alignment. Following the best possible alignment the algorithm was terminated, producing a table of aligned peak sizes, areas, and heights across all samples. Following automated alignment, plots of electropherograms and false gel images can be generated to allow for visual inspection and, if necessary, manual correction of fragment binning prior to downstream analysis. After peak alignment, the T-RFLP profiles of each gene derived from the two different enzyme digestions were combined prior to statistical analysis.

### Statistical analyses

Nitrous oxide fluxes were estimated using HMR (Pedersen et al., 2010), which is available as an add-on package in R (R Core Team, 2015). HMR calculates trace gas flux based on linear or non-linear concentration-time data series as required; linear or non-linear regression was selected manually based on scatter plots of concentration change over time.

The cumulative N_2_O emissions for each of the combinations of year, crop rotation, catch crop and N input used in the experiment were estimated by integrating the N_2_O emissions over the period of observation. To do so, a gamma linear mixed model was adjusted to the N_2_O emissions observed on sampling days in each of the 20 sampling positions represented each year. The model contained a fixed effect representing the combination of year, crop rotation, catch crop, fertilization method and sampling date, and a random component designed to account for the correlations generated by repeated measurements. The integrals over time, representing the cumulative N_2_O emissions in each field plot, were approximated by contrasts (i.e., linear combinations of the model parameters) with coefficients coinciding with the weights of the trapezoidal approximation of the respective integrals, as described in Duan et al. (2017) (Supplementary Material). The analyses were performed with the software R (R Core Team, 2015) using the packages *lme4* for adjusting generalized linear mixed models, and *pairwiseComparisons* (http://home.math.au.dk/astatlab/software/pairwisecomparisons) for making inferences on the contrasts and *post-hoc* analyses. The *p*-values implicitly used in the *post-hoc* analyses were adjusted for multiple comparisons using the false discovery rate (FDR) (Benjamini and Yekutieli, 2001).

Effects of rotations, catch crop, fertilization, and year on gene copy numbers were evaluated by multivariate analysis of variance using the *manova* function in R. Pairwise differences at α = 0.05 were identified by package *lsmeans* with Tukey's multiple comparison test. Bray-Curtis dissimilarities in the T-RFLP profiles were visualized by ordination analysis (non-metric multidimensional scaling, NMDS) using the *vegan* package. The abundances of T-RFs were presented as relative peak areas, and then transformed using Wisconsin double standardization before being supplied to the *metaMDS* function. The ordination was performed using a random start for 100 runs, with 100 iterations in each run. The number of dimensions from one to six was tested, and three dimensions were selected for final analysis with the assistance of scree plots. Following ordination, a test was conducted to find whether there was a correlation between T-RFLP profiles and soil properties.

Soil properties, including ${\text{NH}}_{4}^{+}$ and ${\text{NO}}_{3}^{-}$ concentrations, soil water content, D_p_/D_0_ values, and cumulative N_2_O emissions, were averaged using the trapezoidal rule. A matrix containing these soil properties was fit to the ordination using the *envfit* function with 1,000 permutation tests. Based on the *p*-values of the results, gradients of soil properties that had a significant effect (*p* < 0.05) were shown in the ordination plots using the *ordisurf* function. Ordination and fitting of environmental vectors were performed with T-RFLP profiles of denitrifier genes (*nirK* and *nirS*), N_2_O reduction genes (*nosZ*-I and *nosZ*-II), as well as with a combined profile of all four denitrification genes (*nirK, nirS, nosZ*-I, and *nosZ*-II).

## Results

### Weather conditions in 2011 and 2012

**Figure 1:**
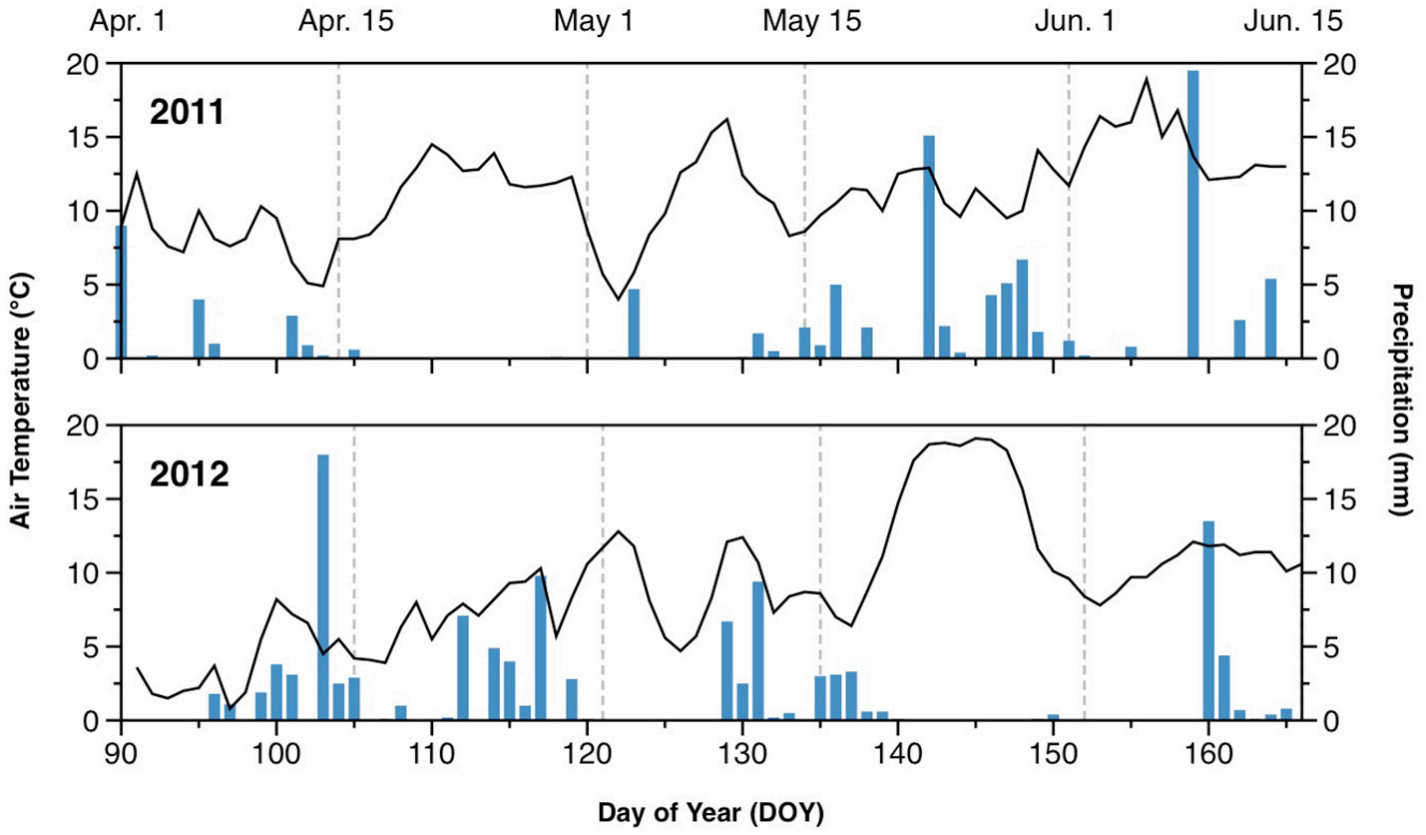
Air temperature (lines) and daily rainfall (bars) during the monitored periods in 2011 and 2012.

The weather in 2011 was generally warmer than in 2012 during the monitoring period, with average temperatures of 11.7°C in 2011 and 9.9°C in 2012 (Figure 1). In particular, there was a cold spell in early April of 2012, with air temperature declining to 1°C. The 2 experimental years also differed with respect to precipitation. The spring of 2011 was drier than that of 2012, with little precipitation before mid-May. In contrast, 2012 had several periods with significant rainfall between early April and mid-May. Average daily precipitation during the monitoring period was 1.4 mm in 2011, and 2.1 mm in 2012.

Soil WFPS varied between 35 and 48% in 2011, and between 29 and 58% in 2012 (Figure S1). Relative gas diffusivity varied between 0.050 and 0.084 in 2011, and between 0.030 and 0.116 in 2012 (Figure S1). Temporal dynamics reflected the distribution of rainfall, with dry periods during April (DOY105–122) in 2011, and in late May and early June (DOY139–159) in 2012. During early spring (April and May), the WFPS was consistently higher, and D_p_/D_0_ lower, in treatments with catch crops (*O4*+*CC*+*N* and *O4*+*CC-N*) in 2012 compared to 2011, whereas WFPS and D_p_/D_0_ were similar in 2011 and 2012 in the two organic rotations without a catch crop (*O4-CC*+*N* and *O4-CC-N*; Figure S1). Higher wetness in 2012 was also indicated for the conventional rotation, *C4-CC*+*N*.

### Nitrogen dynamics

**Figure 2:**
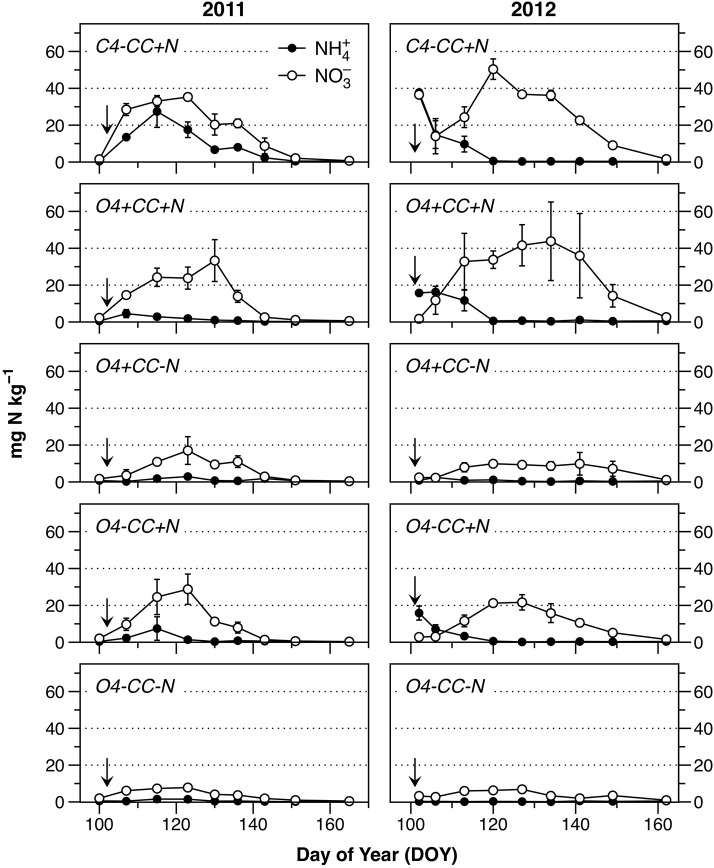
Content of ${\text{NH}}_{4}^{+}$ (filled circles) and ${\text{NO}}_{3}^{-}$ (open circles) in the soil during the monitored periods in 2011 and 2012. Fertilization took place on April 12 (DOY102) in 2011 and April 10 (DOY101) in 2012 (indicated by the arrows). The data represent means of four observations in two replicate plots, while error bars represent standard error (*n* = 2).

The input of N in the form of mineral fertilizer in the conventional system or as digested manure in the organic systems (+*N*), as well as from catch crop residues (+*CC*), was reflected in soil concentrations of ${\text{NH}}_{4}^{+}$ and ${\text{NO}}_{3}^{-}$ (Figure 2). The background levels of both ${\text{NH}}_{4}^{+}$ and ${\text{NO}}_{3}^{-}$ in early spring were low, as seen in the treatment *O4-CC-N*, and in all treatments before N fertilization in 2011 (Figure 2). All treatments showed a similar pattern of mineral N dynamics after fertilization, with ${\text{NH}}_{4}^{+}$ disappearing within 2–4 weeks, and a transient accumulation of ${\text{NO}}_{3}^{-}$. When compared to 2011, the extent of soil ${\text{NO}}_{3}^{-}$ accumulation in 2012 was higher in treatments *C4-CC*+*N* and *O4*+*CC*+*N*, and depletion of soil ${\text{NO}}_{3}^{-}$ occurred later in all treatments.

The accumulation of mineral N was higher in treatments receiving mineral fertilizer (*C4-CC*+*N*) or manure (*O4-CC*+*N, O4*+*CC*+*N*) compared to those with crop residues only (*O4*+*CC-N*). This does not directly reflect the differences in N availability, since the retention time in soil before plant N uptake would have been shorter with a more gradual release of N from catch crop residues. In accordance with this, the N uptake with catch crop residues only (*O4*+*CC-N*) was greater than the uptake with digested manure only (*O4-CC*+*N*) in both years (Table 2); there was little added effect of combining catch crop residues with digested manure (*O4*+*CC*+*N*). The conventional system with NPK fertilizer (*C4-CC*+*N*) had higher plant N uptake than all four organic rotations.

**Table 2:** Nitrogen input (kg N ha^−1^) in catch crop residues and fertilizers, and N content in above-ground plant biomass 2 weeks prior to harvest in late August.

	**Rotation**	**Catch crop N^#^**	**Fertilizer N^#^**	**Plant N uptake**
2011	*C4−CC+N*	–	120	138.6a
	*O4−CC+N*	–	100	78.1b
	*O4+CC−N*	32.3a	0	88.8b
	*O4+CC+N*	40.7a	100	92.0b
2012	*C4−CC+N*	–	120	148.0a
	*O4−CC+N*	–	132	75.1b
	*O4+CC−N*	32.2a	0	85.8b
	*O4+CC+N*	38.0b	132	92.9b

*Data represent means and standard error (n = 2); letters indicate significant differences in plant N uptake (p < 0.05)*.

# *The conventional treatment (C4-CC+N) received NPK mineral fertilizer, while the treatments in the organic system (O4+CC+N and O4-CC+N) received digested manure*.

### N_2_O emissions

**Figure 3:**
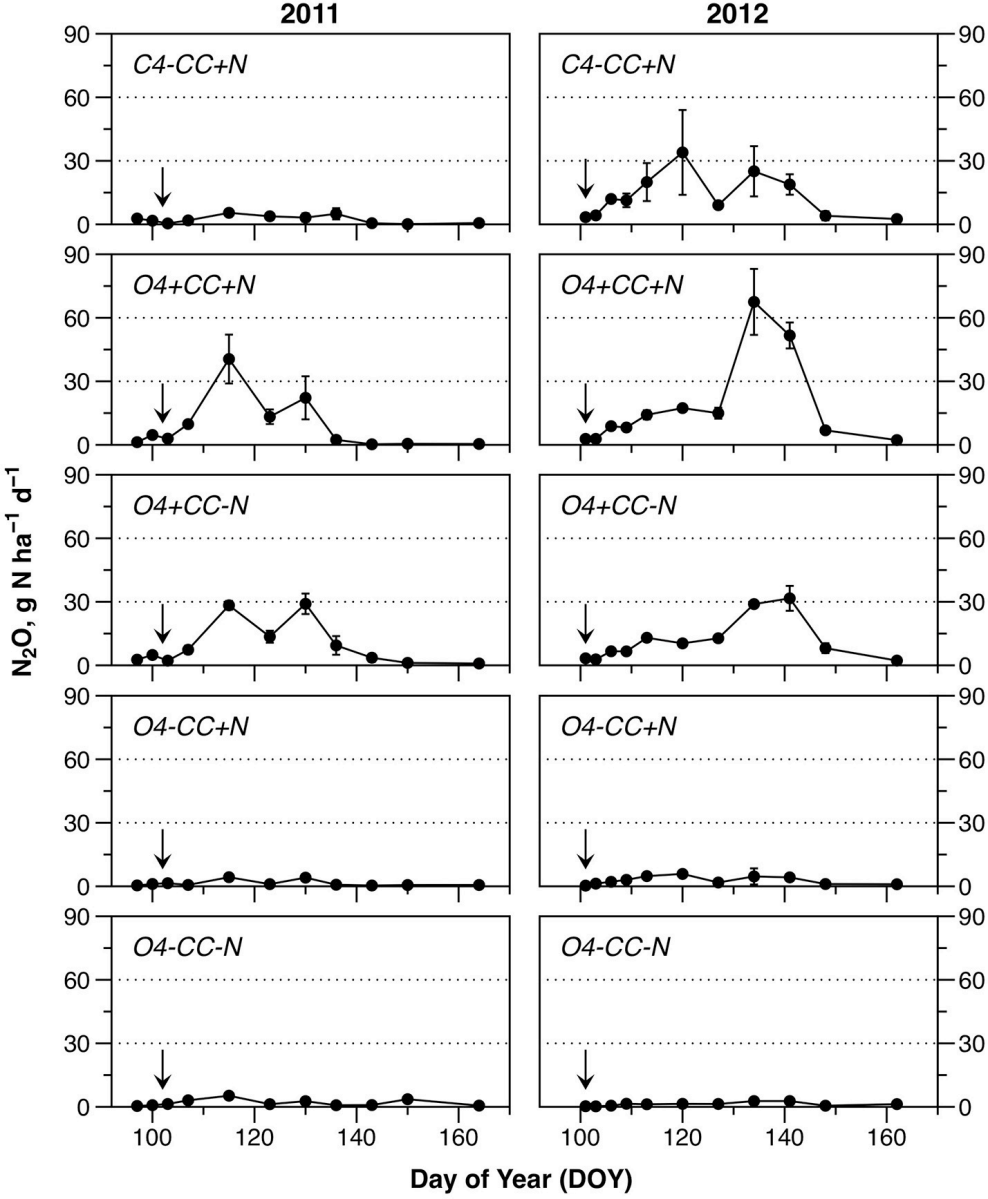
Nitrous oxide emissions during the monitored periods in 2011 and 2012. Fertilization took place on April 12 (DOY102) in 2011 and April 10 (DOY101) in 2012 (indicated by arrows). The data represent means of four observations in two replicate plots, while error bars represent standard error (*n* = 2).

The N_2_O emissions during spring showed several notable trends (Figure 3). Firstly, emissions of N_2_O were higher in both years in rotations with a catch crop (*O4*+*CC*+*N* and *O4*+*CC-N*). In contrast, organic rotations without catch crop incorporation in spring (*O4-CC*+*N* and *O4-CC-N*) had low N_2_O footprints in both years, irrespective of fertilization with digested manure. The conventional rotation without catch crop (*C4-CC*+*N*) showed different patterns in the 2 years, with little or no N_2_O emission in 2011, but substantial emissions in 2012. In both years, the N_2_O emissions in all treatments had returned to the background level by the time of the last sampling. The temporary decline in N_2_O emission rates around DOY125 in 2011, and DOY130 in 2012, coincided with transient cold spells (Figure 3).

Cumulative N_2_O emissions were significantly higher in 2012 than in 2011 for treatments with a catch crop (*O4*+*CC*+*N* and *O4*+*CC-N)*, and for the conventional rotation (*C4-CC*+*N*) (Table 1). In 2011, the cumulative emissions of N_2_O from rotations with catch crop residue incorporation were significantly higher than from rotations receiving N fertilizer only. In 2012, the treatment receiving N in both catch crop residues and digested manure had the highest N_2_O emissions, but the emissions from treatment *C4-CC*+*N* were also significant and similar to those from treatment *O4*+*CC-N*. In 2012, the N_2_O emissions from the organic rotation receiving digested manure only (*O4-CC*+*N*) were again low and only marginally higher than from the unamended reference (Figure 3).

Area-based N_2_O emission factors (EFs) were calculated with reference to N input in catch crop residues and N fertilization; emissions were corrected for background emissions, assumed to be represented by treatment *O4-CC-N*. For treatment *O4*+*CC-N* with catch crop residues as only N input, the area-based N_2_O EF was high in both years (1.7–2.3%) compared to the rotation with both catch crop residue incorporation and digested manure (*O4*+*CC*+*N*) at 0.4–0.7%. The EF for treatment *O4-CC*+*N* receiving digested manure was consistently low. In contrast, the N_2_O EFs for treatment *C4-CC*+*N* receiving mineral fertilizer differed in the 2 years, with no increase in N_2_O emissions in 2011 and 0.7% in 2012. Yield-scaled EFs were calculated with reference to the N content in plant biomass harvested in each of the experimental treatments in August 2011 and August 2012, respectively (Table 2). Yield-scaled EFs were higher in 2012 compared to 2011 (Table 1).

### Abundances of denitrifier genes

**Figure 4:**
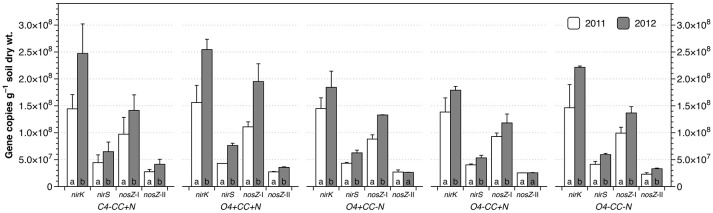
Abundance of *nirK, nirS, nosZ*-I, and *nosZ*-II genes in different cropping systems in 2011 and 2012. The bars represent the average in each treatment, and error bars show standard error (*n* = 2). Within each treatment, genes marked with the same letters are not significantly different at α = 0.05 between 2011 and 2012.

The abundances of *nirK* genes were 1.38–1.56 × 10^8^ and 1.79–2.54 × 10^8^ copies g^−1^ dry soil in 2011 and 2012, respectively, and around three times higher than the copy numbers of *nirS* genes (Figure 4). The *nosZ*-I genes were significantly more abundant than *nosZ*-II genes, with copy numbers ranging from 8.82–11.1 × 10^7^ copies g^−1^ soil in 2011 to 1.18–1.95 × 10^8^ copies g^−1^ soil in 2012, which was three to four times the abundance of *nosZ*-II genes. Within each treatment, the abundances of all four genes increased significantly from 2011 to 2012, except for *nosZ*-II genes in *O4*+*CC-N* and *O4-CC*+*N*. In contrast, there were no significant effects of the experimental variables (rotation, catch crop, N addition or interactions) with respect to gene abundances within each year, as determined by multivariate analyses of variance. The average ratios of *nir* to *nos* gene copy numbers (*nir*/*nos* ratios) for all treatments were approximately 1.56 in both years, and there were no significant difference (*p* > 0.05) across treatments and/or years.

### Denitrifier community structure

**Figure 5:**
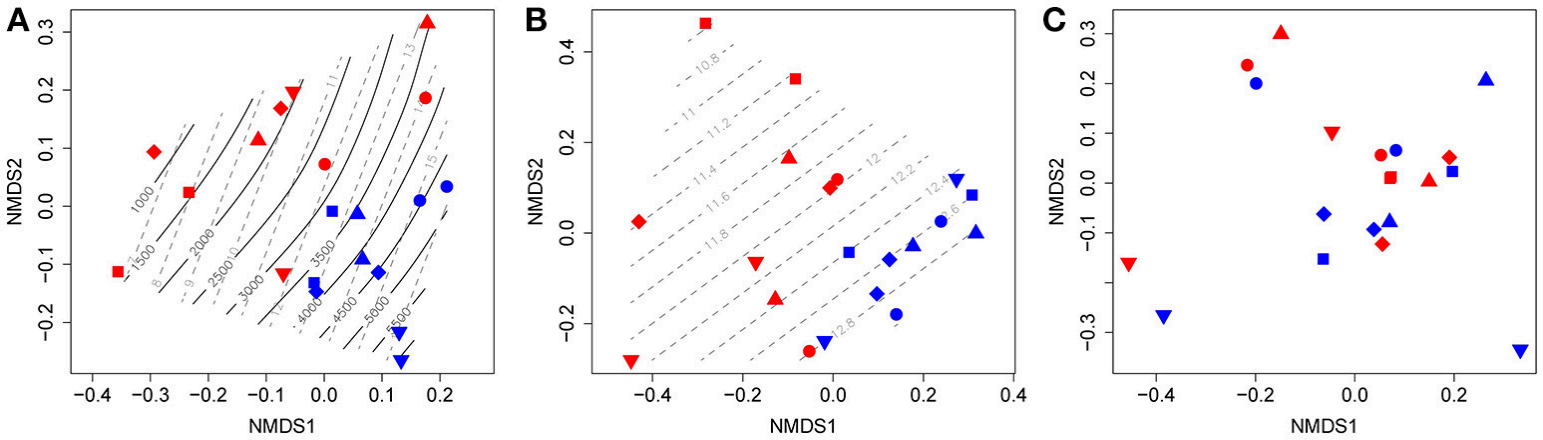
Non-metric multi-dimensional scaling (NMDS) ordination of T-RFLP profiles for **(A)** collective denitrification genes (*nirK, nirS, nosZ*-I, and *nosZ-*II); **(B)** nitrite-reduction genes (*nirK* and *nirS*); and **(C)** N_2_O-reduction genes (*nosZ*-I and *nosZ*-II). Each point represents the T-RFLP profile of one field plot. Environmental vectors that were significantly correlated to shifts in T-RFLP profiles (*p* < 0.05) were fitted to the ordination and presented as gradients: black solid lines represent cumulative N_2_O emissions (g N_2_O-N ha^−1^), and gray dashed lines represent average soil ${\text{NO}}_{3}^{-}$ content (mg N kg^−1^ soil). ∙ : *C4-CC*+*N*; ■: *O4-CC-N*; ♦: *O4-CC*+*N*; ▴: *O4*+*CC-N*; ▾: *O4*+*CC*+*N*. Red points represent samples from 2011, and blue points those from 2012.

The ordination of the combined T-RFLP profiles of *nirK, nirS*, and *nosZ* clade I and II genes show two distinct clusters, representing samples from 2011 and 2012, which reveals a shift in community structure between years (Figure 5A). These changes correlated strongly to ${\text{NO}}_{3}^{-}$ concentrations (*p* = 0.042), as well as cumulative N_2_O emissions (*p* = 0.035). Gradients of D_p_/D_0_ also partly described this inter-annual variation; however, the correlation was not significant (*p* = 0.114). Samples were more scattered in 2011 compared to 2012, suggesting less overall heterogeneity in 2012. Separate ordination analyses of T-RFLP profiles for nitrite reduction genes (*nirK* and *nirS*; Figure 5B) and N_2_O reduction genes (*nosZ* clade I and II; Figure 5C) show that the changes in community structure between years were associated with denitrifiers carrying *nir* genes rather than *nos*-harboring N_2_O-reducers. A significant shift along the gradient of ${\text{NO}}_{3}^{-}$ concentrations was also observed for the *nir* communities (Figure 5B; *p* = 0.032). In contrast, no correlation between community structure and environmental variables was found for N_2_O-reducing communities, and there was no effect of management on the structure of any of the communities in either year.

## Discussion

### Oxygen supply and demand

WFPS is often used as a proxy for soil O_2_ status (Chen et al., 2008), and approximately 60% WFPS has been considered to be an upper limit for well-aerated soil conditions (Linn and Doran, 1984). Some studies, however, suggest that relative gas diffusivity, D_p_/D_0_, is a better predictor of N_2_O emissions from intact (Petersen et al., 2008) as well as repacked soil (Balaine et al., 2013), and in the present study both metrics of soil O_2_ status were therefore calculated (Figure S1). Both WFPS and D_p_/D_0_ indicated that soil O_2_ availability was lower during April and May of 2012 (WFPS ~55%, D_p_/D_0_ ~0.04) compared to 2011 (WFPS ~45%, D_p_/D_0_ ~0.06) in the conventional rotation, and in the two organic rotations with catch crops. In accordance with this, the N_2_O emissions were also significantly higher in 2012 in treatments *C4-CC*+*N* and *O4*+*CC*+*N*, whereas the difference was not significant in *O4*+*CC-N* (Table 1). Generally, emissions of N_2_O occurred at bulk soil conditions that should not support N_2_O emissions, i.e., < 60% WFPS (Linn and Doran, 1984; Balaine et al., 2013). In a related study, Chirinda et al. (2010) found evidence for soil compaction at 0–5 cm depth in *C4-CC*+*N*, which may have increased soil water-retention and restricted the O_2_ supply in this treatment, but does not explain N_2_O emissions in the two organic rotations where, instead, organic hotspots may have been the main source. Parkin (1987) demonstrated that nearly all denitrification activity in a soil core was associated with a single decaying leaf. The depletion of O_2_ around residues was demonstrated by Højberg et al. (1994) using an O_2_ microsensor, and by mapping of O_2_ distribution with planar optodes (Kravchenko et al., 2017). Kravchenko et al. (2017) further showed that plant residues absorbed water in order to equilibrate with the soil water potential, thereby attaining 4–10 times more water by volume than the surrounding soil. Parkin (1987) calculated that a 160-μm water film would be sufficient to develop anaerobic conditions at surfaces of decomposing plant material, and hence water absorption may represent a barrier for O_2_ supply allowing denitrification and N_2_O emissions to occur even in well-aerated soil. In accordance with this, Li et al. (2016) found consistent N_2_O emissions from leguminous catch crop residues incubated at 40, 50, and 60% WFPS, and denitrification was shown to be the main source of N_2_O at all three soil water levels. This was also the case in a manipulation experiment with intact soil from organic crop rotations incubated at water potentials of −10, −30, and −100 hPa (Petersen et al., 2013a). Thus, in soil environments with organic hotspots, denitrification can occur over a wide range of soil moisture conditions—what matters is the balance between O_2_ supply and O_2_ demand, which could also account for much of the variation in N_2_O emissions observed in the present study.

### Nitrogen availability

Degradable organic carbon and O_2_ limitation are only two of the three requirements for denitrification, the third being the electron acceptors ${\text{NO}}_{3}^{-}$ or ${\text{NO}}_{2}^{-}$. Background levels of ${\text{NO}}_{3}^{-}$ in the soil were low (Figure 2), but increased instantaneously with NPK fertilization, and more gradually with digested manure and catch crop residues as N source. A phase of net N immobilization may occur when crop residues and liquid manure are applied to soil. For example, Trinsoutrot et al. (2000) found that rapeseed incorporation resulted in net N immobilization for *c*. 2 weeks, and Sung et al. (2010) reported little N immobilization from rye, but substantial net N mineralization from hairy vetch. This implies that N_2_O emissions during the initial stage of decomposition will depend on soil ${\text{NO}}_{3}^{-}$ availability for several days, as reported by Petersen et al. (1996) in a study of cattle manure hotspots. The supply of ${\text{NO}}_{3}^{-}$ from the soil will rapidly decline as a result of decreasing concentration gradients, and sustained N_2_O emissions therefore depend on mineralization and nitrification of N input via manure or crop residues. The digested manure used in this study contained only 2.6–3.6% DM, and thus most of the liquid phase would have infiltrated the bulk soil, along with dissolved C and N. The intimate contact between soil and manure probably enhanced microbial N immobilization (Sørensen and Jensen, 1995), thereby reducing N availability for nitrification and denitrification, resulting in lower N_2_O emissions. Kong et al. (2017) incubated ^15^N-labeled residues of white clover in soil mesocosms and found that the enrichment of N_2_O increased gradually during a 2-week period (though much less so when residues had been treated with a nitrification inhibitor to prevent nitrification of mineralized N). Generally, residue quality will determine the extent of net N mineralization from decomposing residues (Li et al., 2016).

Soil mineral N dynamics indicated that plant N uptake was delayed in 2012 compared to 2011. This was probably related to a difference in soil temperature, since by DOY140 (mid-May) the sum of plant-growing degree days, calculated according to Léon (1992), were 219 and 131 in 2011 and 2012, respectively (Figure S2). The longer residence time for mineral N in the soil probably increased the potential for N_2_O emissions by increasing the average soil ${\text{NO}}_{3}^{-}$ availability.

Positive effects of catch crops on yields are normally seen when access to mineral N in fertilizers or manure is suboptimal (Li et al., 2015; Marcillo and Miguez, 2017). However, the relationship between N input and plant N uptake in this study was weak and suggested that plant availability of the N supplied in digested manure and residues was relatively low. An earlier study from the same long-term crop rotation experiment found that differences in yield of winter wheat could not be explained by labile N pools (potentially mineralisable N, microbial biomass N) alone (Petersen et al., 2013b). Instead, a multiple regression analysis showed that total N and depth of the A horizon, and cumulative N input during the previous 12 years, all contributed significantly to N availability. It implies that available N, and N_2_O emissions, are not exclusively derived from the most recent input of manure and residues, and that long-term effects of management influence N_2_O emissions *via* net N mineralization, nitrification and denitrification.

### Denitrifier community dynamics

Genetic potential for net N_2_O emissions was indicated by *nir*/*nos* gene copy ratios > 1 across all treatments and both years. The increase in N_2_O emissions in 2012 was corroborated by a significant increase in the abundances of *nirK* and *nirS* genes, suggesting that the size of the community matters (Hallin et al., 2009). However, there was no difference in *nir*/*nos* ratios between the 2 years, and no correlation was found between *nir*/*nos* ratios and N_2_O emissions. This lack of correlation indicates a more complex regulation of the N_2_O balance than mere gene copy numbers, and that subsequent regulations of gene transcription and enzymatic activities are important in the shorter term (Röling, 2007). Expression of *nosZ* may be impaired by low pH (Liu et al., 2014), and in most cases N_2_O reductase loses activity if exposed to O_2_ (Thomson et al., 2012). However, Højberg et al. (1994) did not find evidence for a decrease in pH around a decaying clover leaf, and O_2_ supply was probably lower, not higher, in 2012 compared to 2011 (cf. Figure S1). In contrast, the accumulation of ${\text{NO}}_{3}^{-}$ around decomposing residues or manure-saturated soil volumes would have been greater in 2012, resulting in lower ratios of metabolizable C vs. ${\text{NO}}_{3}^{-}$, which is known to increase the N_2_O:N_2_ product ratio (Benckiser et al., 2015). The increase in N_2_O emissions from 2011 to 2012 was also associated with changes in the collective communities carrying *nir* and *nos* genes (Figure 5). Although net N_2_O emissions were the result of a balance between N_2_O production and consumption, the inter-annual shift observed for the collective denitrifier communities was only found for communities carrying *nirS*, but not *nirK* nor *nosZ*, genes (Figure 5; Figure S3). This suggests that *nirS*-type denitrifiers accounted for the higher N_2_O emissions in 2012. Different responses of *nirS*- and *nirK*-type denitrifiers is consistent with the concept that the two variants respond differentially to environmental factors (Hallin et al., 2009; Jones and Hallin, 2010; Braker and Conrad, 2011).

Organic or mineral N fertilizers, and catch crop residue decomposition, have the potential to modify denitrifier communities through effects on soil ${\text{NO}}_{3}^{-}$ and O_2_ availability, and metabolizable carbon (Hallin et al., 2009; Enwall et al., 2010; Tatti et al., 2015). In the present study, however, a statistically supported response in denitrifier gene abundances to nitrogen management was not observed. Furthermore, management appeared to have limited effect on the composition of denitrifier communities, even after more than a decade with the same crop rotation (Figure 5). The main difference was instead between year 2011 and 2012, which was associated with a difference in ${\text{NO}}_{3}^{-}$ and, to some extent, O_2_ availability. Higher ${\text{NO}}_{3}^{-}$ availability in general in 2012 could be explained by delayed plant uptake, as discussed above, and the O_2_ supply was reduced because of higher precipitation. Under such conditions, with more anoxic periods and fluctuating soil O_2_ status, the denitrifiers have an advantage compared to obligate aerobic microorganisms. The higher ${\text{NO}}_{3}^{-}$ availability combined with lower O_2_ availability in the first month after tillage and fertilization, and the availability of metabolizable C, probably together stimulated the activity and growth of *nirS* denitrifiers in 2012 compared to 2011, leading to the inter-annual shift in community composition and elevated N_2_O emissions. Hence, the pressure caused by the year-to-year differences in abiotic parameters was stronger than selective pressure from management for these functional groups. This suggests that climatic factors rather than management could impact future N_2_O emissions from denitrification and climate feedbacks.

## Perspectives

Both area-based and yield-scaled N_2_O emission factors increased in all treatments between 2011 and 2012, although treatments and cropping histories were identical. The annual application of 100 kg N ha^−1^ or more in digested manure resulted in no or barely measurable emissions of N_2_O in both 2011 and 2012, whereas N_2_O emissions in treatments with catch crop residue incorporation were high in both years despite lower N input (cf. Tables 1, 2). The NPK treatment, in contrast, showed low and high emissions in 2011 and 2012, respectively. These observations challenge the methodology of the Intergovernmental Panel on Climate Change (IPCC, 2006), in which emissions are estimated from N input only. The IPCC methodology is a statistical approach and acknowledges the diversity of soil conditions by defining a large uncertainty range (0.003–0.03) for the default N_2_O emission factor of 0.01. However, the patterns of N_2_O emissions and soil characteristics observed here across five experimental rotations and 2 years suggest that there may be scope for better predictions of N_2_O emissions by taking site-specific conditions into account. This should include soil physical properties and precipitation, but also the amount and quality of organic C input as a potential driver for denitrification in organic hotspots. Given that catch crop residues, by the inclusion of above-ground parts, will often have a higher degradability and lower C:N ratio compared to roots and stubble of harvested crops (Trinsoutrot et al., 2000), and that incorporation takes place in spring where soil water content is often higher than at harvest, there is an urgent need to consider catch crop residues as a driver for N_2_O emissions, and search for mitigation options.

## Conclusions

Rotations with a catch crop during winter had significantly higher N_2_O emissions after spring incorporation than rotations without catch crop, and stimulated N_2_O emissions more consistently than addition of N, either as mineral fertilizer or digested manure. Contrary to our original hypothesis, there was limited evidence for a positive interaction between crop residues and N fertilizer application, whereas the importance of rainfall for N_2_O emissions from mineral N fertilizer was confirmed. This indicates an important role of crop residues in regulating N_2_O emissions from sandy soils, where transformations of residue-derived N probably took place in organic hotspots with O_2_ limitation caused by intense turnover of degradable residue carbon. The abundance of denitrifier genes increased from 2011 to 2012, and the inter-annual shift in community composition was associated with gradients in ${\text{NO}}_{3}^{-}$ availability. The changes in both the community size and structure were correlated to higher N_2_O emissions in 2012 compared to 2011. However, management differences between the five rotations had limited effect on the abundance and structure of nitrite- and N_2_O-reducers. Together these results suggest that rotations with catch crops significantly stimulated N_2_O emissions from agricultural soil, but had limited effect on the genetic potential for denitrification and N_2_O reduction.

## Author contributions

SP designed the study and organized the field experiment. Y-FD performed molecular analyses in collaboration with SH and AP. CJ developed the R package used for T-RFLP alignment. RL provided consultation on statistical analysis of N_2_O emission data. Y-FD and SP wrote the first draft of the manuscript. All authors contributed to the development of the manuscript.

### Conflict of interest statement

The authors declare that the research was conducted in the absence of any commercial or financial relationships that could be construed as a potential conflict of interest.
