# Supplementary material for: Catch Crop Residues Stimulate N2O Emissions During Spring, Without Affecting the Genetic Potential for Nitrite and N2O Reduction
Source: Front Microbiol. 2018 Nov 2;9:2629. doi: 10.3389/fmicb.2018.02629 (PMC6225543; doi:10.3389/fmicb.2018.02629)
Supplement: Supplementary file 1 [file Data_Sheet_1.pdf]

Supplementary Material

Catch crop residues stimulate N_2O emissions during spring, without affecting the genetic potential for nitrite and N_2O reduction

Yun-Feng Duan [1], Sara Hallin [2], Christopher M. Jones [2], Anders Priemé [3], Rodrigo Labouriau [4], Søren O. Petersen [1]

1. Department of Agroecology, Aarhus University, Tjele, Denmark
2. Department of Forest Mycology and Plant Pathology, Swedish University of Agricultural Sciences, Uppsala, Sweden
3. Section of Microbiology, Department of Biology, University of Copenhagen, Copenhagen, Denmark
4. Department of Mathematics, Aarhus University, Aarhus, Denmark

Correspondence to:

Søren O. Petersen

sop@agro.au.dk

Alignment of terminal restriction fragments (T-RFs) using R package *fragalign*

T-RFs derived from soil samples in the present study were aligned using an in-house R package *fragalign*. The principals involved in the alignment algorism are described in the manuscript. Here, we list the parameters used in the analysis in the present study. The R source code of the package can be downloaded as a separate file.

Parameters:

```
color = "B"         # Fragment color.
frag.min = 50       # Minimum fragment size.
frag.max = 700      # Maximum fragment size.
noise.SD = 3        # Threshold for detecting noise peaks; default is 3, reduce to include more peaks.
norm = "h"          # Noise filtering option; h = filter noise based on peak height; a = filter based on peak area.
gaussR = 1.5        # Gaussian peak contrasts; blurs peaks - for PeakScanner, should be 1-3; GeneMapper should be 30-35.
merge.a = 0.04      # Merging parameter a - most useful for merging peaks with shoulders, increase to increase merging -
                    # set to 0 to turn off merging; default is 0.04 from Vähamää et al., 2007.
merge.b = 0.6       # Merging parameter b - default from Vähamää.
merge.c = 3.5       # Merging parameter c - default from Vähamää.
e = 0.05            # Alignment parameter e - default from Vähamää is 0.013; increase to allow greater range of fragments
                    # to be matched.
f = 0.8             # Alignment parameter f - default from Vähamää is 1.
g = 3.5             # Alignment parameter g - default from Vähamää is 3.5; absolute allowable size to be matched in bp.
min.n = 2           # Minimum number of fragments to keep sample - otherwise tossed.

# Call the fragment alignment function
out <- fragalign(fileName, color, frag.min, frag.max, noise.SD, norm, gaussR, merge.a, merge.b, merge.c, e, f, g, min.n)
```

Table S1. Primers and thermal cycling conditions for quantitative amplification of denitrifier genes.

Gene	Primers	Thermal cycling conditions for qPCR	Reference
nirK	F1aCu: ATCAT GGTSC TGCCG CG<br>R3Cu: GCCTC GATCA GRTTR TGGTT	Initial denaturation at 95 °C for 10 min; 5 cycles of 95°C for 15 sec, 63°C for 30 sec (decreased by 1°C per cycle), and 72°C for 30 sec; 40 cycles of 95°C for 15 sec, 58°C for 30 sec, 72°C for 30 sec, 83°C for 15 sec, followed by plate reading.	Hallin and Lindgren (1999)
nirS	Cd3aF: AACGY SAAGG ARACS GG<br>R3cd: GASTT CGGRT GSGTC TTSAY GAA	Initial denaturation at 95 °C for 10 min; 5 cycles of 95°C for 15 sec, 65°C for 30 sec (decreased by 1°C per cycle), 72°C for 30 sec; 40 cycles of 95°C for 15 sec, 60°C for 30 sec, 72°C for 30 sec, 83°C for 15 sec, followed by plate reading.	Throbäck et al. (2004)
nosZ Clade I	nosZ_1F: CGYTG TTCMT CGACA GCCAG<br>nosZ_1R: CGSAC CTTST TGCCS TYGCG	Initial denaturation at 95 °C for 10 min; 5 cycles of 95°C for 15 sec, 65°C for 30 sec (decreased by 1°C per cycle), 72°C for 30 sec; 40 cycles of 95°C for 15 sec, 60°C for 30 sec, 72°C for 30 sec, 83°C for 15 sec, followed by plate reading.	Henry et al. (2006)
nosZ Clade II	nosZ-II_F: CTXGG XCCXY TKCAY AC<br>nosZ-II_R: GCXGA RCARA AXTCB GTRC	Initial denaturation at 95 °C for 10 min; 40 cycles of 95°C for 30 sec, 54°C for 60 sec, 72°C for 60 sec, 82°C for 15 sec, followed by plate reading.	Jones et al. (2013)

References

Hallin, S., and Lindgren, P.-E. (1999). PCR Detection of Genes Encoding Nitrite Reductase in Denitrifying Bacteria. *Applied and Environmental Microbiology* 65(4), 1652-1657.

Henry, S., Bru, D., Stres, B., Hallet, S., and Philippot, L. (2006). Quantitative detection of the *nosZ* gene, encoding nitrous oxide reductase, and comparison of the abundances of 16S rRNA, *narG*, *nirK*, and *nosZ* genes in soils. *Applied and Environmental Microbiology* 72(8), 5181-5189. doi: 10.1128/aem.00231-06.

Jones, C.M., Graf, D.R.H., Bru, D., Philippot, L., and Hallin, S. (2013). The unaccounted yet abundant nitrous oxide-reducing microbial community: A potential nitrous oxide sink. *The ISME Journal* 7(2), 417-426. doi: 10.1038/ismej.2012.125.

Léon, J. (1992). Crop Growth Rates and Durations of Spring Barley Cultivars as Affected by Varied N Supply and Seeding Rates. *Journal of Agronomy and Crop Science* 169(1-2), 1-8. doi: doi:10.1111/j.1439-037X.1992.tb01179.x.

Throbäck, I.N., Enwall, K., Jarvis, Å., and Hallin, S. (2004). Reassessing PCR primers targeting *nirS*, *nirK* and *nosZ* genes for community surveys of denitrifying bacteria with DGGE. *FEMS Microbiology Ecology* 49(3), 401-417. doi: 10.1016/j.femsec.2004.04.011.

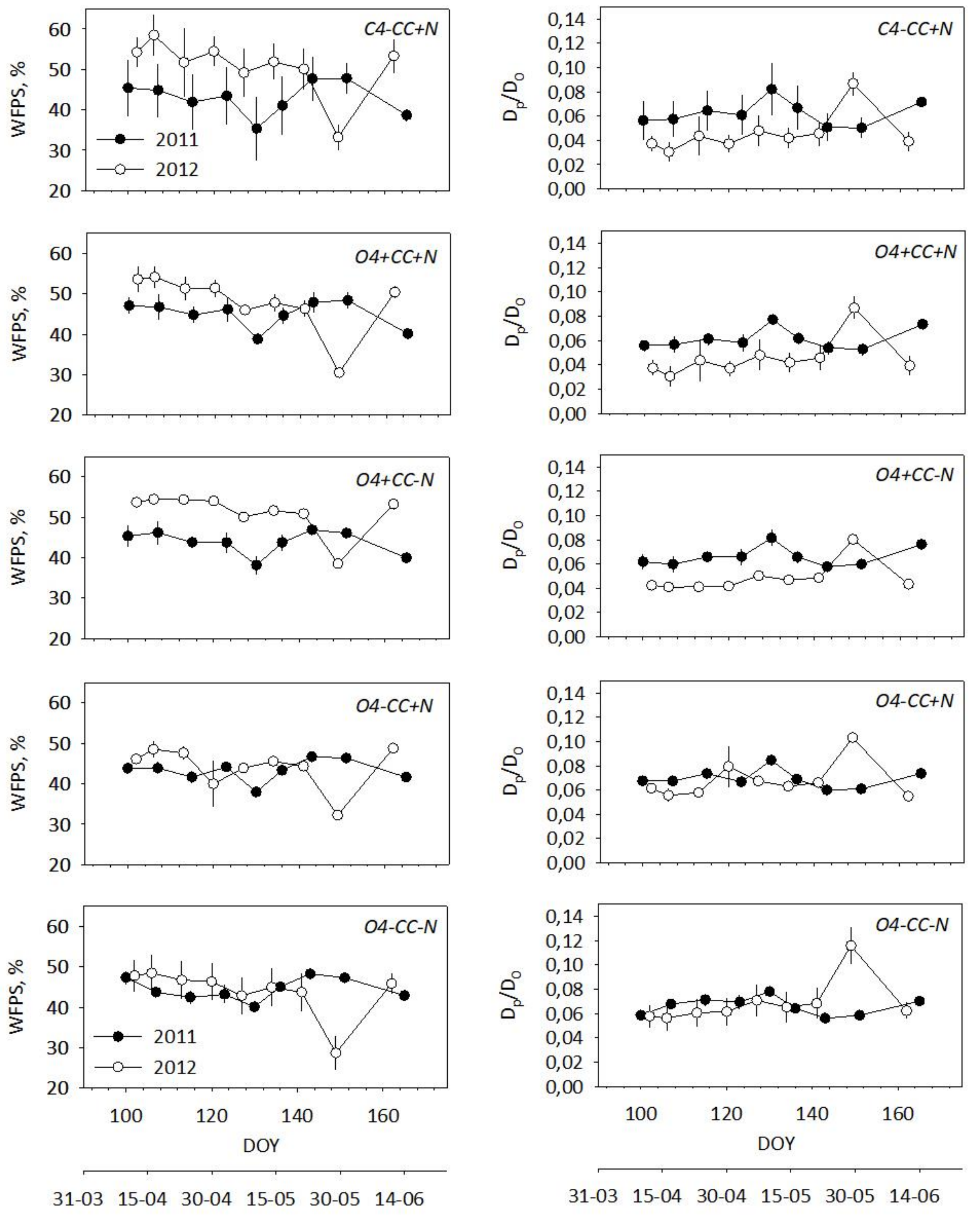

Figure S1. Soil water-filled pore space (WFPS) and relative soil gas diffusivity (D_p/D_0), as defined by Moldrup et al. (2005).

Moldrup, P., Olesen, T., Yoshikawa, S., Komatsu, T., and Rolston, D.E. (2005). Predictive-descriptive models for gas and solute diffusion coefficients in variably saturated porous media coupled to pore-size distribution: II. Gas diffusivity in undisturbed soil. *Soil Science* 170(11), 854-866. doi: 10.1097/01.ss.0000196768.44165.1f.

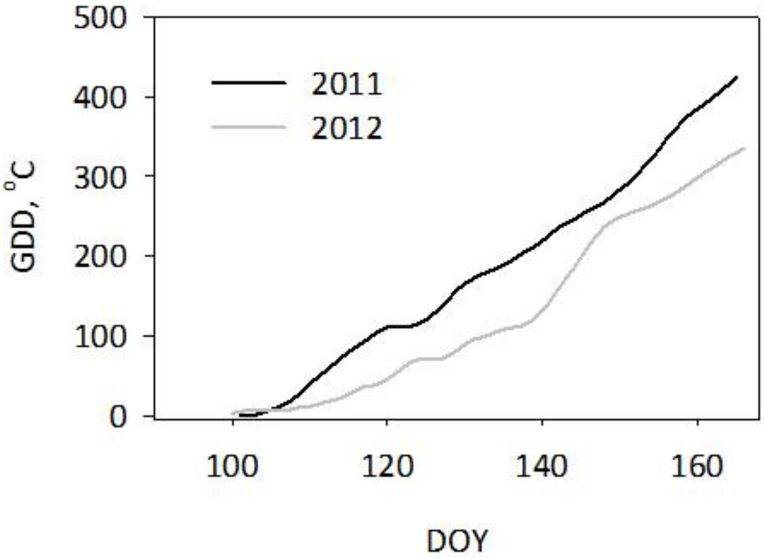

Figure S2. Growing degree days, GDD, calculated as: $GDD = \sum \max[T_d, T_{base}] - T_{base}$, where T_d is daily average temperature and T_{base} is a base temperature of 5°C (Léon, 1992).

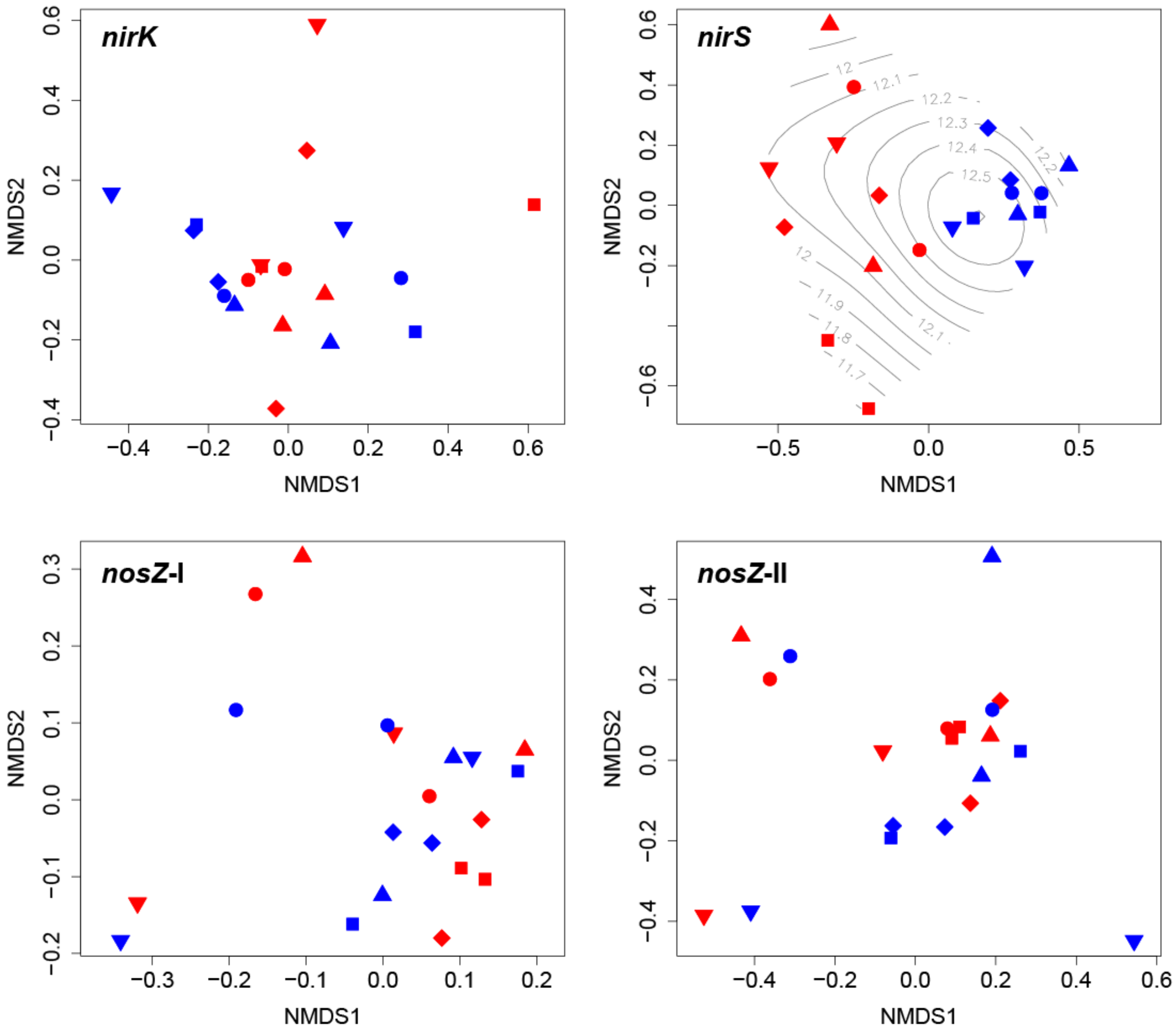

Figure S3: Non-metric multi-dimensional scaling (NMDS) ordination of T-RFLP profiles for *nirK* , *nirS*, *nosZ*-I, and *nosZ*-II genes. Each point represents the T-RFLP profile of one replicate plot. Average soil NO_3^- concentrations (mg N kg^{-1} soil) are fitted to the ordination of *nirS* genes ($p < 0.05$) and presented as gradients. ●: *C4-CC+N*; ■: *O4-CC-N*; ◆: *O4-CC+N*; ▲: *O4+CC-N*; ▼: *O4+CC+N*. Red points represent samples from 2011, and blue points those from 2012.
